# Spectroscopic Methods Applied in Food Quality Determination

**DOI:** 10.3390/foods15101818

**Published:** 2026-05-20

**Authors:** Xiaohong Wu

**Affiliations:** School of Electrical and Information Engineering, Jiangsu University, Zhenjiang 212013, China; wxh419@ujs.edu.cn

## 1. Introduction

Food quality and safety are the fundamental cornerstones supporting the sustainable development of the global food industry, public health protection, and the maintenance of consumer trust and market fairness. With the continuous expansion of the food supply chain and the increasing diversification of consumer demands, efficient, accurate, and green quality detection technologies have become an urgent need for the whole process of food production, processing, circulation, and sales [1]. However, traditional food quality analysis methods, such as conventional physical and chemical detection, are mostly limited by cumbersome sample pretreatment, toxic reagent consumption, destructive testing, long detection cycles, and high laboratory dependence [2,3]. These drawbacks prevent them from adapting to the requirements of real-time monitoring, on-site rapid screening, and non-destructive testing in modern intelligent food quality control scenarios.

In recent years, spectroscopic technologies have emerged as powerful and promising tools for food quality determination, relying on their inherent advantages of non-destructive testing, rapid responses, high precision, and easy integration with automated and intelligent systems [4,5]. A variety of advanced spectroscopic modalities, including near-infrared spectroscopy, mid-infrared spectroscopy, Raman spectroscopy, hyperspectral imaging (HSI), and terahertz spectroscopy, have been deeply explored and widely applied in the field of food analysis [6,7]. These techniques can capture the molecular vibration, spectral reflection, and absorption characteristics of food samples synchronously, thereby realizing the acquisition of multi-dimensional information of food components and properties without damaging the sample structure or affecting product value [8]. More importantly, when coupled with advanced chemometric algorithms and machine learning models, including support vector machines (SVMs), adaptive boosting (Adaboost), and linear discriminant analysis (LDA), spectroscopic tools can effectively mine the hidden correlation between high-dimensional spectral data and food quality indicators, enabling simultaneous qualitative identification and quantitative analysis of food nutritional components, geographical origin, freshness, and oxidation degree [9,10].

This Special Issue of Foods, titled “Spectroscopic Methods Applied in Food Quality Determination”, aims to collect research achievements on the innovative integration of spectroscopic technologies and chemometric methods for food quality assessment. The collected research fully demonstrates the latest technological progress, practical application value, and industrialization potential of spectroscopic technologies combined with chemometrics in modern food analysis, and it also provides reliable technical solutions and theoretical support for whole-process quality control in the food industry. This editorial systematically summarizes the core research findings of the published papers in this Special Issue and presents some representative studies to facilitate a better understanding of the issue.

## 2. Overview of Published Contributions

Malondialdehyde (MDA) is a critical marker for lipid oxidation and meat quality deterioration. Bhandari et al. (Contribution 1) established an ultrafast kinetic fluorogenic method using 2-thiobarbituric acid (TBA) as a probe to quantify MDA in ground beef. The whole assay only takes 6 min, greatly shortening the detection time compared with traditional methods. The study also validated the effects of air exposure, washing, and cooking on MDA content, providing practical guidance for meat handling and processing. This simple and robust protocol shows high potential for industrial rapid screening.

Mehany et al. (Contribution 2) developed a sustainable analytical strategy for olive and sunflower oils by employing NIR spectroscopy coupled with the stepwise decorrelation method and ordinary least squares (OLS) regression. The simplified models achieved reliable prediction of pigments, antioxidant capacity, and core sensory descriptors, including bitter, fruity, rancid and pungent notes. The method performed well even under deep-frying conditions, supporting rapid quality monitoring in the edible oil sector with high accuracy and low computational cost.

Wu et al. (Contribution 3) proposed a novel hybrid model combining adaptive boosting (Adaboost) and common vectors linear discriminant analysis (CLDA) for the geographical origin classification of red jujube. Using NIR spectroscopy with Savitzky–Golay filtering, the method effectively solved the “small sample size” problem and achieved extremely high identification accuracy. This work offers a dependable technical approach for the origin traceability and quality authentication of jujube products.

Zhang et al. (Contribution 4) applied hyperspectral imaging (HSI) combined with machine learning algorithms to realize non-invasive evaluation of pear fruit quality. Adopting Fast Detrend-Standard Normal Variate (FD-SNV) pretreatment and Competitive Adaptive Reweighted Sampling (CARS), the models precisely forecasted various important indicators such as pH value, firmness, ripeness, and color. The backpropagation neural network (BPNN) model reached over 99% accuracy in variety classification, and multi-variety modeling further improved model robustness, supporting industrial online detection of fruit quality.

Tangorra et al. (Contribution 5) used a miniaturized handheld NIR spectrometer coupled with a hybrid LDA-SVM model to classify commercial milk types, including fresh milk, mountain milk, extended shelf-life milk, and TSG hay milk. The method realized rapid, non-destructive, and on-site discrimination of milk based on production systems and heat treatments. This portable solution supports real-time authenticity verification at retail points and helps prevent food fraud in the dairy supply chain.

To achieve accurate moisture monitoring for carrot slices during freeze-drying, Wang et al. (Contribution 6) integrated near-infrared spectroscopy (NIRS) with low-field nuclear magnetic resonance (LF-NMR) to realize the rapid and non-invasive measurement of total moisture, free water and bound water. Aiming to identify the most effective modeling schemes for both full-moisture and low-moisture systems, this work carried out a comprehensive comparison of diverse spectral preprocessing techniques, sample partitioning algorithms, feature extraction methods and machine learning models. On the basis of the optimized parameters, a staged moisture prediction model was established, which offers a steady and dependable technical approach for online non-destructive moisture surveillance in the freeze-drying production of fruits and vegetables.

## 3. Conclusions

This Special Issue fully presents the cutting-edge applications and methodological innovations of spectroscopic techniques integrated with chemometrics and machine learning in food quality determination. The included studies cover a wide range of food matrices, such as meat, edible oils, fruits, dairy products and agricultural products, and solve practical problems including rapid detection of quality markers, non-destructive evaluation of product properties, geographical origin traceability and on-site authenticity identification. These research achievements verify the high efficiency, non-destructiveness, accuracy and industrial adaptability of spectroscopic methods and provide solid technical support and theoretical reference for green, intelligent, and whole-process quality control in the food industry. With the continuous optimization of spectral devices, modeling algorithms and data fusion strategies, spectroscopic detection technologies will further expand their application scenarios and play a more critical role in ensuring global food quality and safety.

## Data Availability

Data sharing is not applicable.
